# Postnatal Cardiac Autonomic Nervous Control in Pediatric Congenital Heart Disease

**DOI:** 10.3390/jcdd3020016

**Published:** 2016-04-15

**Authors:** Ineke Nederend, Monique R. M. Jongbloed, Eco J. C. de Geus, Nico A. Blom, Arend D. J. ten Harkel

**Affiliations:** 1 Department of Biological Psychology, Faculty of Behavioral and Movement sciences, VU Amsterdam, Van der Boechorststraat 1, 1081 BT Amsterdam, The Netherlands; eco.de.geus@vu.nl; 2 EMGO+ Institute for Health and Care Research, VU Medical Center Amsterdam, Van der Boechorststraat 7, 1081 BT Amsterdam, The Netherlands; 3 Department of Pediatric Cardiology, LUMC University Medical Center, Albinusdreef 2, 2333 ZA Leiden, The Netherlands; N.A.Blom@lumc.nl (N.A.B.); A.D.J.ten_Harkel@lumc.nl (A.D.J.H.); 4 Department of Cardiology and Anatomy & Embryology, LUMC University Medical Center, Albinusdreef 2, 2333 ZA Leiden, The Netherlands; M.R.M.Jongbloed@lumc.nl

**Keywords:** autonomic nervous system, congenital heart disease, children, heart rate variability

## Abstract

Congenital heart disease is the most common congenital defect. During childhood, survival is generally good but, in adulthood, late complications are not uncommon. Abnormal autonomic control in children with congenital heart disease may contribute considerably to the pathophysiology of these long term sequelae. This narrative review of 34 studies aims to summarize current knowledge on function of the autonomic nervous system in children with a congenital heart defect. Large scale studies that measure both branches of the nervous system for prolonged periods of time in well-defined patient cohorts in various phases of childhood and adolescence are currently lacking. Pending such studies, there is not yet a good grasp on the extent and direction of sympathetic and parasympathetic autonomic function in pediatric congenital heart disease. Longitudinal studies in homogenous patient groups linking autonomic nervous system function and clinical outcome are warranted.

## 1. Introduction

Congenital heart defects are the most common congenital defects, affecting around 90 per 10,000 newborns [1]. Nowadays, for most of these defects, adequate catheter interventions, surgical correction or repair is available, which has resulted in a dramatic improvement of survival. Ninety percent of children born with a congenital heart disease (CHD) survive into adulthood [2]. Despite good survival in childhood, a substantial amount of patients with a CHD who reach adulthood suffer late complications such as pulmonary hypertension [3] and arrhythmias and die from heart failure, sudden cardiac death or other cardiac problems [4,5]. It is not known how heart function is kept at bay during childhood, which patients develop problems later in life and how to prevent or reverse this process. Chronic changes in cardiac autonomic control to compensate for hemodynamic alterations due to CHD or due to surgical intervention may play a role. Thus, assessment of cardiac autonomic function may provide insight in future disease progression. Increased sympathetic activity and decreased parasympathetic activity (which can be due to multiple causes) is strongly associated with myocardial dysfunction. Enhanced sympathetic activity plays a significant role in the progression of heart failure and it is very plausible that it also plays a role in the long term sequelae in congenital heart disease, including eventual fibrosis.

A fairly large body of research exists on cardiac autonomic function in adult heart disease. Altered autonomic function is found in various patient groups; for example, in cardiac failure [6] and patients after coronary artery bypass [7], decreased heart rate variability (HRV) is found. Altered function of this system in cardiac patients usually entails increased sympathetic and decreased parasympathetic activity and is associated with an increased risk of cardiac events and sudden cardiac death in patients with known cardiovascular disease [8,9], but also in persons without a history of cardiovascular disease [10]. However, it is still uncertain whether this dysfunction is part of the pathophysiology, mainly a compensation mechanism, caused by surgical procedures or a combination of these. Recent work has shown that autonomic regulation in fetuses with CHD already differs from healthy fetuses [11]. In a healthy fetus, mean heart rate will decrease and heart rate variability (HRV) will increase with gestational age [11,12]. In affected fetuses, decreased HR was found to be paired to decreased HRV. Subgroup analysis revealed that especially fetuses with hypoplastic left heart syndrome showed decreased HRV, and this was not observed in the groups with transposition of the great arteries and tetralogy of Fallot. Differences were detectable as early as 19 weeks of gestational age. A possible mechanism of the altered autonomic nervous system (ANS) development in CHD might be alterations in cerebral blood flow [13] and therewith brain development [14] caused by structural changes in circulation. Brain volume and head circumference is smaller in fetuses with CHD compared to controls [15,16] and cerebrovascular resistance is lower [17]. In healthy fetuses, autonomic control seems to be associated with maternal exercise behavior [18] and this effect is maintained after birth [19].

As opposed to adults, children have little or no comorbidities. Therefore, they constitute a clean group to study ANS function and unravel its role in etiology. In this narrative review, we aim to summarize current knowledge on autonomic function in children with a congenital heart defect after birth. Before doing so, we will briefly describe the main structure and function of the ANS, ways to measure ANS and healthy maturation of the ANS.

## 2. Autonomic Nervous System

The term “autonomic nervous system” was coined by Langley in 1898. The main function of the ANS is to ensure homeostasis and coordinate bodily functions in response to a dynamic external and internal environment. The ANS can be subdivided into two branches; the sympathetic and the parasympathetic branch. Coarsely taken, the first is responsible for the “fight or flight” response whereas the second branch has a key role in the “rest and digest” state of the body. At any moment, both these branches are active at the same time but the balance of the two depends on the specific demand of the particular situation requiring a sympathetic or parasympathetic (or equal) dominance of the two systems. The sympathetic nervous system (SNS) is responsible for increasing heart rate, contractility of the cardiac muscle, blood pressure, epinephrine secretion, sweat production and breathing frequency in order to make the body ready for action. The preganglionic sympathetic fibers leave the central nervous system from the thoracic and lumbar regions of the spinal cord. These preganglionic fibers employ acetylcholine (ACh) as primary neurotransmitter and synapse onto the sympathetic ganglia (sympathetic trunk) which is located close to the spinal cord. The longer postganglionic neurons employ norepinephrine (NE) as their primary neurotransmitter. NE acts on α-1 adrenergic or β-1 or β-2 adrenergic receptors. Preganglionic neurons also directly innervate the adrenal medulla, which releases NE and epinephrine (E) into the blood stream. Circulating NE is converted into E which has affinity for binding to β-2 receptors causing vasodilatation and increase of heart rate and contractility. α-1 receptors cause vasoconstriction by acting on smooth muscles. β-1 and β-2 receptor activation will cause increased contractility of the ventricles and will increase heart rate by accelerating phase 4 of the pacemaker action potential. An increased activity of the SNS is an important and powerful mechanism of the body to compensate altered hemodynamics, e.g., due to heart disease. However, chronic exposure to enhanced levels of NE concentrations can in time cause maladaptive and even detrimental effects to the cardiovascular system and organs [20]. Based on this knowledge, prescription of beta blockers is currently the cornerstone of treatment of heart failure. Additionally, Angiotensin Converting Enzyme inhibitors and diuretics to counteract the SNS-mediated increase in angiotensin I, aldosterone and fluid retention are used to tackle this “heart failure cascade” and thereby lead to significantly improved survival [21].

**Figure 1:**
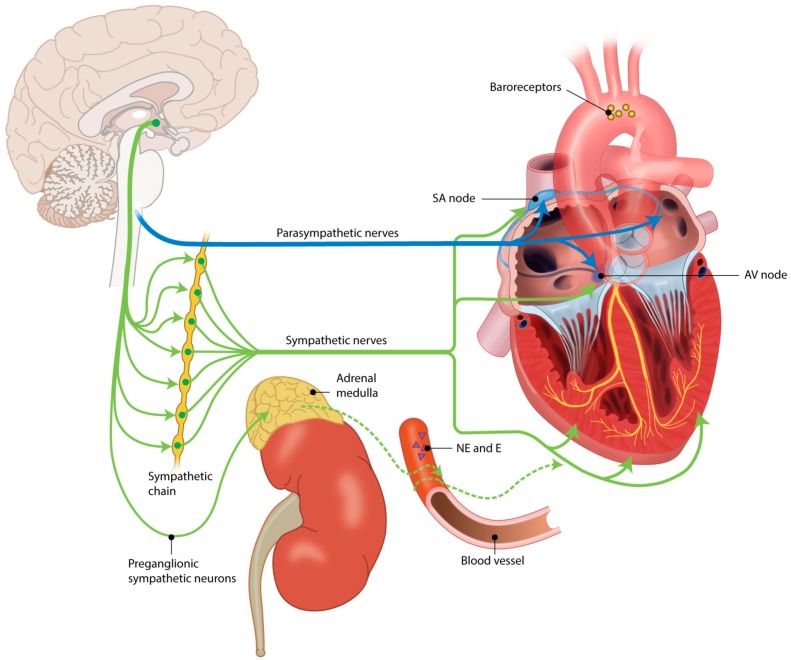
Schematic of autonomic cardiovascular control.

The parasympathetic nervous system (PNS) effectuates the opposite of the SNS; *i.e.*, a decrease in heart rate, breathing frequency, *etc.* The vagus nerve (cranial nerve X) originates from the medulla oblongata, which serves as the center for cardiovascular reflexes. The long preganglionic fibers of the PNS (originating from the vagus nerve) are the primary source of parasympathetic innervation of the heart (and other organs). Parasympathetic ganglia generally lie close or even in the effector organs. ACh is the primary neurotransmitter of both the pre- and postganglionic PNS fibers. Receptors on the postganglionic fibers are of the nicotinic subtype whereas the receptor subtypes on the target organ are one of the five muscarinic acetylcholine receptor subtypes (M1–M5). The predominant cardiac subtype is M2. Parasympathetic innervation is more dense in the atria, the sinoatrial and in the atrioventricular node than in the ventricles [22]. The functional role of this ventricular innervation has not yet been elucidated. Baroreceptors register blood pressure (stretch of the arterial wall) and send this information to the medulla oblongata which will respond in order to maintain pressure. Arterial baroreceptors are located in the carotid sinuses and aortic arch, and cardiopulmonary baroreceptors in the atria, ventricles and pulmonary vessels. Higher pressure will increase baroreceptor firing and decrease sympathetic activity, resulting in a decrease in blood pressure. Baroreceptor-mediated blood pressure control mainly buffers short term blood pressure variations. In the long term, blood pressure is regulated by the endocrine system. A schematic of autonomic cardiovascular control can be found in Figure 1.

## 3. Postnatal Measurement of Cardiac Autonomic Control

A technique for assessing cardiac sympathetic innervation is a metaiodobenzylguanidine (MIBG) scan. This scintigraphy employs a radiolabeled molecule, similar to noradrenaline; MIBG labeled to iodine-123 (I^123^-MIBG). Cardiac sympathetic innervation can be quantified as the ratio of MIBG uptake in the heart to MIBG uptake in a reference area, e.g., mediastinum (H/M ratio) [23]. A low H/M ratio indicates low sympathetic innervation. MIBG scintigraphy is a cardiac neurotransmission imaging technique to assess presynaptic reuptake and storage whereas at the effector level, cardiac ANS activity is assessed. The local sympathetic innervation as measured by MIBG uptake is only a part of that limb as circulating catecholamines also play a role.

Autonomic nervous system function is complex and difficult to measure. Gold standards for measuring cardiac ANS activity are unfortunately also the most invasive methods. Invasive techniques include measurement of norepinephrine (NE) regional spillover, microneurography (direct measurement of action potentials in the nerve), microdialysis (measurement of acetylcholine (Ach) in the dialysate sample in which the probe is designed to mimic a blood capillary and measures passive diffusion in the tissue) and pharmacological blockade (heart rate and diastolic blood pressure are measured both before and after pharmacological blockade of SNS or PNS (by e.g., metropolol/propranolol or atropine) [24]. These techniques are highly to moderately invasive and therefore not preferable, certainly not in pediatric studies.

There are also several non-invasive techniques available for the measurement of cardiac autonomic nervous control; including analysis of heart rate and HRV which will be discussed in more detail below. Another non-invasive measurement is baroreflex sensitivity (BRS) by measuring beat-to-beat changes in heart rate and blood pressure e.g., by a finger cuff (Finapres). BRS can also be evaluated in a lower body negative pressure test. The patient’s lower body is placed in a tube where negative pressure is applied which reduces venous return while changes in blood pressure and heart rate are monitored. The valsalva maneuver follows the same reasoning but in this test venous return is reduced by increasing the intrathoracic pressure by performing forced expiration to an obstruction. By the use of two skin electrodes, (hand palm) skin conductance level can be used as measure for SNS activity. Sweat glands will increase productivity with increased SNS. The subsequent increase in conductance of the skin is used as a measure of SNS activity as sweat glands are innervated by the sympathetic but not the parasympathetic nerves. The focus in research has been largely on heart rate and blood pressure. However, these variables represent an unknown mixture of sympathetic and parasympathetic input. Since health outcomes for sympathetic and parasympathetic hyperactivity are different, it is important to distinguish between the two.

Pediatric studies most often employ measurement of HRV for the measurement of ANS activity. For most HRV analysis, an electrocardiogram is the only necessity. The existence of beat-to-beat variation in pulse has been known from 1733 when Stephen Hales described that heart rate varies with respiration [25]. Ever since those days, physicians have had a great interest in heart rate variability and its relationship with disease. After the invention of the “physician’s pulse watch” by Floyer in the early 1700s, changes in pulse rate could be accurately assessed. This portable clock enabled him to tabulate both respiration and pulse. The first clinical relevance of HRV was described in 1965 by Hon and Lee [26] who noted that a change in HRV preceded fetal distress. HRV is now a frequently used tool to evaluate autonomic control [27] although some groups argue that HRV primarily depends on heart rate and therefore cannot be used in any simple way to assess nervous activity on the heart [28].

**Figure 2:**
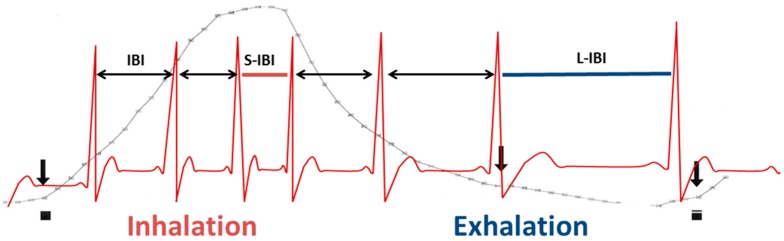
Respiratory sinus arrhythmia. Electrocardiogram in red, respiration in grey. IBI (black double headed arrows); inter-beat interval; the time between two consecutive R-peaks in the ECG. RSA; respiratory sinus arrhythmia; calculated as longest heart period during exhalation (L-IBI) minus the shortest heart period during inhalation (S-IBI).

**Figure 3:**
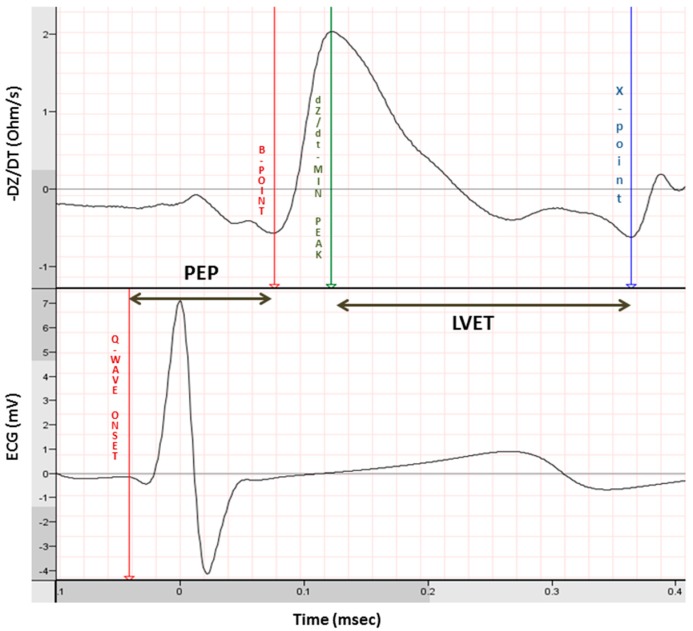
Systolic time intervals including PEP. PEP; pre ejection period. LVET; left ventricular ejection time.

An overview of the most used HRV variables is given in Table 1. In general, HRV provides measures for PNS but not SNS activity. HRV can be divided into time domain and frequency domain analysis. For these HRV analysis, normal-to-normal (NN) interbeat intervals are used. That is, only heartbeats originating from the sinus node are used and ectopic beats should be removed. Respiratory sinus arrhythmia (RSA) provides a good PNS measure and can be measured with only electrocardiography (RMSSD, HF) or by combining electrocardiography with respiratory signal recording (pvRSA; see Figure 2). Coupled to respiration rate, firing of motor neurons in the nerves ambiguous and the sympathetic nuclei is phasically inhibited and exited. This phenomenon is caused by connections between the nuclei that control the respiratory generator in the pre-Bötzinger and Bötzinger complexes and parasympathetic and sympathetic motor neurons. These connections modulate the release of neurotransmitters in such a way that during inspiration the release of NE is increased and the release of Ach is decreased. During expiration the opposite occurs; release of NE is decreased and the release of Ach is increased. As a result, heart rate increases with inspiration and decreases with expiration and RSA is generated [29]. Changes in hyperpolarization of the sinus node in response to parasympathetic outflow occur within hundreds of milliseconds. However sympathetic outflow modulates the depolarization speed only on the scale of seconds and therefore is too slow to follow the phasic respiratory-coupled changes. Hence, the SNS does not contribute to RSA [30]. In the case of high PNS activity, thus many action potentials per second, the effect of the phasic inhibition and excitation will be more pronounced compared to when there is less PNS activity. As a result, RSA will be higher when there is more PNS activity. Respiration rate and tidal volume have to be considered when interpreting (changes in) RSA as those also have an independent influence on HRV. However, during approximately similar respiration, HRV provides a measure for parasympathetic outflow to the heart. Other HRV measures (*TP*, *ULF*, *VLF*, *LF*) are comparable to respiratory sinus arrhythmia, but these lower frequencies in heart rate are also influenced by sympathetic input. A higher respiratory sinus arrhythmia indicates higher PNS activity. The LF/HF ratio is a frequently used measure of SNS activity but has met with much controversy [24]. When employing impedance cardiography in addition to the electrocardiogram, a non-invasive measure of SNS can be derived: the pre-ejection period (PEP) [31]. The impedance cardiogram is computed by sending an alternating current through the thorax. The impedance (*i.e.*, complex resistance) will change over time due to respiration and the amount of blood in the thorax. The first derivative of this signal (*i.e.*, change in impedance) is the impedance cardiogram (Figure 3, upper graph) and can be used to derive systolic time intervals.

The PEP is a measure of contractility and is defined as the time delay between the start of the electrical depolarization of the ventricles (Q-onset) and the start of the actual outflow of blood into the aorta (B-point) (Figure 3). Functional ventricular parasympathetic innervation is only proven in canine models. There is increasing evidence for parasympathetic innervation in human ventricles but the functionality remains to be elucidated [22]. Therefore, the PEP provides us with a pure measure of SNS activity. A shorter PEP indicates higher SNS activity. The T-wave amplitude, by reflecting sympathetic effects on repolarization can be used in addition to the PEP in order to characterize sympathetic activity non-invasively [32]. A lower T-wave indicates higher SNS activity.

## 4. Cardiac Autonomic Nervous System in Healthy Maturation

In normal maturation of the ANS, several factors are of influence. In a recent review, Eyre *et al.* [33] give an overview of studies concerning the effect of age, gender and weight status. Most, but not all, studies in children found a positive correlation between age and HRV (in both time and frequency domain) measures, indicating a progressive increase in cardiac PNS activity with age. This pattern of development seems to be most progressive in infancy, continues more gradually in early childhood and even more so in late childhood. Heragu *et al.* [34] (not included in the review) also found a significant age-related increase in HRV in 45 healthy children aged 3 weeks to 16 years. The effect found of gender on ANS in childhood is not consistent [33]. Some studies report no gender differences [35], whereas others report higher HRV in males compared to females [36,37]. Resting values for heart rate, pre ejection period and respiratory sinus arrhythmia in different body positions are described in 3097 children aged 5–7 years old by van Dijk *et al.* [38]. Significant sex differences were found for all three measures (heart rate and pre ejection period being higher for girls and respiratory sinus arrhythmia lower for girls) but not in all body postures. A better understanding of the specific time course and nature of the normal development of ANS in childhood would be of great importance in order to better discriminate between health and disease and to be able to intervene as early as possible.

## 5. Cardiac Autonomic Control in Pediatric Congenital Heart Disease

To summarize the current knowledge on cardiac autonomic control in pediatric congenital heart disease we below present a narrative review of the extant literature on this topic. The Pubmed database was used as the primary source for the literature search. Search terms used were “autonomic nervous system” [MeSH] AND “congenital heart disease” [MeSH]. The MeHS entry terms were also entered as free text in order to find missed recent papers that were not yet indexed in the Pubmed database. English papers from 1965 onwards only were included and additional filters on age (child: birth–18 years) and language (English) were employed. We focused on structural defects; research concerning inherited rhythm disorders or channelopathies were excluded. Also, case studies were excluded. The search yielded a total of 197 papers. After screening on title and abstract, 55 were identified as potentially relevant and five were added after screening of reference lists. Thirty-four were included in the final review. Twenty-six studies were excluded because they included adults, did not concern congenital heart defects or did not measure ANS activity. Table 2 concisely summarizes the preoperative, immediate postoperative and long term postoperative findings per CHD. Table 3 gives an overview of all studies included; six studies on transposition of the great arteries, three on Tetralogy of Fallot, six on atrial- or ventricular septal defect, four on univentricular heart, five on coarctation and 10 on various CHD.

In cardiac disease, ANS control may adapt in order to compensate for changed hemodynamics. This change is usually characterized by an increased sympathetic and/or a decreased parasympathetic tone and works as a powerful compensation mechanism. Activation of arterial- and cardiopulmonary baroreceptors will change due to altered hemodynamics in the heart and blood vessels and may, with prolonged exposure to increased pressures, reset to a higher value. For instance, in response to heart failure, ANS activity can change. Since the early 90s, the idea has arisen that the deterioration of heart failure had in part a neurohormonal explanation [39]. Before this time, progress in heart failure was believed to be due solely to hemodynamic stress, triggered by the initial injury, e.g., in prolonged left ventricular failure, pressure and preload of the left atrium will rise. As a result, the resistance in the pulmonary vascular system (the afterload of the right ventricle) will increase and may eventually lead to right ventricular failure. The neurohormonal explanation includes increased activity of the SNS and the renin-angiotensin system. This is a powerful compensatory mechanism to deal with altered hemodynamics in rest and/or during exercise, depending on the severity of heart failure, but prolonged exposure of NE in chronic heart failure can cause damage to myocytes and cause β-receptor down regulation that will ultimately diminish heart function [40]. In cardiac failure, resting levels of the SNS measured by regional NE spillover can increase to as much as 50 times which is comparable to the SNS response to maximal exercise in healthy man [41]. Sympathetic over activation may cause leakage of the ryanodine receptor 2 channel, located on the sarcoplasmic reticulum. The subsequent calcium leakage causes a decrease in cardiac contractility [42]. Furthermore, evidence suggests that an increased sympathetic tone is arrhythmogenic [43] while high parasympathetic levels have a cardioprotective effect [44]. Parasympathetic tone increases the electrical stability of the heart [45,46,47,48] but if it is delayed after potentials as a result of the calcium leakage through ryanodine channels, it may potentially cause arrhythmia. The maladaptive responses that can be directly related to increased SNS activity have contributed to the insight that beta-blockers are beneficial in the treatment of heart failure. By decreasing plasma E and NE levels, these agents likely modulate the maladaptive sympathetic responses and slow the heart failure cascade.

Different mechanisms are involved in various heart defects and the alterations in hemodynamics and ANS control are expected to be specific to the defect at hand. Also, the type of eventual intervention employed for repair (e.g., transcutaneous or via sternotomy) may have a substantial influence on the ANS. Altered ANS function may be due to pulmonary hypertension, systemic hypertension, heart failure, pressure- or volume overload following a (or multiple) cardiac malformation(s). Consequently, hemodynamics and thus ANS activity might also change following repair. Unfortunately, some studies do not take this into account and group a variety of different defects and patients before and after repair together for analysis of ANS function (see Table 3). Also, potential changes in pulmonary functional status must be taken into account. Especially following sternotomy, pulmonary function may be worsened [49] and tidal volume and breathing frequency might change and effect HRV.

### 5.1. Ventricular and Atrial Septal Defect

Atrial and ventricular septal defects (ASD and VSD respectively) are relatively common with a prevalence of 13 and 41 per 10,000 live births respectively [50]. The hemodynamic changes that accompany these defects are dependent on the size and location of the defect, the resistance in pulmonary and systemic vasculature and the atrial and ventricular pressures. In uncomplicated ASD, oxygenated blood is shunted from the left to the right atrium. The resulting volume overload may induce enlargement of the right atrium, right ventricle and pulmonary artery. The ventricular volume overload may cause a decrease in HRV via baroreceptors and stretch of the sinoatrial node [51]. Also, the respiratory fluctuation in atrial pressure may be altered or at least be proportionally less compared to healthy subjects, causing a reduction in HRV [52,53]. Several authors studied pediatric ASD patients preoperatively [54,55,56] and found decreased HRV (both time and frequency domain) compared to healthy controls. Additionally, Massin and colleagues [55] found a negative correlation between HRV and right atrial pressure and between HRV and end diastolic right ventricular pressure. Finley *et al.* [53] and Bialkowski *et al.* [56] studied their patients a few months postoperatively. Compared to controls, Finley found higher supine SDNN (indicating higher PNS tone) after repair compared to before but this difference disappeared while in upright position. Bialkowski and colleagues compared patients before and after transcutaneous or surgical ASD repair and found that in the transcutaneous repaired, HRV increased early (1 month) after repair and increased further after 3 months. In contrast, in the surgical group, HRV reduced to below preoperative values shortly after but recovered to higher compared to preoperative values 3 months after.

In uncomplicated VSD, the defect also causes a left-to-right shunt, potentially causing enlargement of the left atrium and left ventricle because of increased blood return to the left side of the heart. The left ventricular volume overload may via baroreceptors change autonomic control and decrease HRV [51]. Hata *et al.* [52] compared ASD and VSD patients preoperatively. Compared to ASD patients, HF power in respiratory frequency band was significantly higher in VSD patients indicating higher parasympathetic tone in VSD patients. No difference was found in respiratory rate, LF, TP nor LF/HF. Kul Yum [57] studied HRV in a group of VSD patients during catheterization and found lower HF and LF in a subgroup of patients with high pulmonary artery pressure compared to patients with normal pressure. After having been on cardiopulmonary bypass for the correction of the septal defect, cardiac ANS activity and HRV can be altered compared to healthy persons. In a subgroup (28 ASD and 3 VSD) analysis, Ohuchi and colleagues [58] found a reduction of HF (log transformed) and baroreceptor sensitivity 1 month after surgery, indicating a drop in parasympathetic tone. One year after surgery, they were back to preoperative values.

In conclusion, preoperatively, HRV seems to be reduced in ASD patients, indicating lower PNS activity compared to healthy controls. There is not enough evidence from VSD cohorts to draw a conclusion on this issue. Postoperatively, HRV seems to normalize, although long term evidence is lacking and, also, age of intervention might play a role as the time of exposure to high pressures may have an influence.

### 5.2. Transposition of the Great Arteries

In transposition of the great arteries (TGA), the aorta arises from the right- and the pulmonary artery from the left ventricle, resulting in two parallel circulations leading to cyanotic heart disease. Reported prevalence is 2.3 per 10,000 live births [50]. Hemodynamics and therewith clinical presentation is dependent on the degree of mixing of the two parallel circulations via the ductus arteriosus, foramen ovale and presence of anomalies causing shunts (e.g., VSD). TGA requires neonatal surgical intervention. As sympathetic nerves course along the origin of the great arteries to find their way to the heart, they may be injured during surgery at this site. Operated patients are at increased risk of ventricular tachycardia due to scar tissue or repolarization instability following altered cardiac autonomic control or polymorphic ventricular tachycardia due to right ventricular dysfunction. Kondo *et al.* [67] show that cardiac sympathetic nerves are denervated in 4 patients shortly (<1 month) after arterial switch operation (ASO) for correction of TGA, measured by MIBG uptake. Before surgery, all children had positive MIBG uptake. As a control group, they also measured this in four children operated for a ventricular septal defect (VSD) and found normal innervation an all four shortly after surgery. Furthermore, 47 patients were measured late after ASO and 32% showed absent MIBG uptake. Cardiac nerves were re-innervated in almost all patients that were operated in early infancy (≤55 days). In contrast, this was seen in only half of the patients after late operation. Furthermore, they found that patients who did show re-innervation, were more likely to have normal exercise capacity. There is one more study that evaluated ANS function before reconstruction, they found decreased HF power in 15 patients, suggesting decreased parasympathetic activity [68]. A few weeks after surgery (up to 8 weeks), there was no difference in resting HF power (PNS activity) but patients did show a delayed recovery of HF power back to baseline after feeding. In another study by the same group, HRV was measured during a task (block stacking) 3 years after surgery. Again, mean resting values were not different between patients and controls but PNS reactivity (decrease in HF while doing the task) was lower [69]. In a third study on the same subject, authors do report a lower resting and recovery (HF power increase after feeding) HF power in patients 2 weeks after operation, at 8 weeks the difference had disappeared [70]. In a study on 22 patients without arrhythmia and operated in the neonatal period, a significantly higher SDNN, rMSSD, pNN50, TP and VLF was found in awake patients compared to controls at 12-188 months after surgery suggesting higher parasympathetic tone [71]. During sleep, no differences in HRV were detected. Authors conclude that patients have a predominant parasympathetic tone although HF is not significantly different between groups. Falkenberg *et al.* found that cardiac NE reuptake is impaired while parasympathetic function measured by baroreceptor sensitivity was not different between patients after ASO and controls [72]. In conclusion, sympathetic activity seems to be intact preoperatively while parasympathetic activity is found to be reduced. Postoperatively, evidence on parasympathetic activity is equivocal. Sympathetic cardiac activity seems to be decreased shortly after repair but is able to recover late after surgery, especially when operation is conducted before the age of 55 days.

### 5.3. Univentricular Heart

Different surgical procedures are available for the management of univentricular heart, all are palliative in nature. ANS control may be altered by abnormal load or heart failure before operation and after, ANS may be altered due to the surgical intervention, heart failure and changed hemodynamics. Although the overall incidence is low, arrhythmia is an important cause of morbidity in patients with a total cavopulmonary connection [85]. In the long term, arrhythmia often develops in these patients leading to severe heart failure and risk of sudden cardiac death. HRV could be an effective tool to detect this complication in an early stage [74]. As a result of surgery or as a result of pre-existing anatomical issues, there may be sinus node pathology that overlays the autonomic control. Madan *et al.* [73] compared ANS activity in patients at two different stages of univentricular palliation; bidirectional Glenn shunt (BDG) and total cavopulmonary connection (Fontan completion). Before operation, there was no difference in time and frequency domain HRV between the two groups. One month after surgery, RMSSD was higher in the BDG group compared to Fontan patients and 8 months later, the coefficient of variation was higher in this group. Unfortunately, this study group did not include a healthy control group. Ohuchi *et al.* [75] studied a large group of 63 patients after Fontan procedure and 44 controls. PNS activity was evaluated by HRV, BRS, and the change in heart rate after cholinergic blockade. SNS activity was evaluated by means of plasma NE, MIBG ratio and the change in heart rate after isoproterenol infusion. They found that all ANS indexes were lower in patients compared to controls except for heart rate change to isoproterenol. NE was higher in patients. Their results indicate a decreased PNS activity and sympathetic innervation. There was no relationship between ANS indices and age, age at operation, hemodynamics, type of operation nor time after operation. Maximal exercise testing revealed lower exercise capacity, higher resting heart rate and lower maximal heart rate and heart rate reserve in patients compared to controls. A significant correlation was found between heart rate reserve and all ANS indices except for H/M ratio. Butera *et al.* [76] studied 24 h holter monitoring of 39 patients with total or partial cavopulmonary or atriopulmonary connection and two control groups; 18 healthy volunteers and 16 patients after biventricular repair for their CHD (defects not specified). Patients after total cavopulmonary connection had most aberrant HRV and were significantly different from both healthy controls and the CHD group with biventricular repair. In conclusion, no studies have been conducted on ANS function before surgery compared to healthy controls. Postoperatively, HRV is reduced in patients compared to controls. Both parasympathetic and sympathetic activity seem to be reduced in these patients.

### 5.4. Coarctation of the Aorta

Coarctation of the aorta (CoA) is a local narrowing of the descending aorta, typically located distal from the left subclavian artery at the insertion of the ductus arteriosus and has a prevalence of 4.4 in 10,000 live births [50]. CoA results in an increased LV afterload, causing increased pressures and potentially dilation of the left heart. Also, it causes high blood pressure in the upper- and low blood pressure in the lower part of the body. ANS in uncorrected CoA can be altered because of low blood pressure to the lower part of the body, including to the kidneys. Consequently, the renin-angiotensin system will be activated to increase systemic blood pressure. On the other hand, the increased pressure, decreased elastic properties [86] and secondary flow patterns [87] in the aortic arch may alter function of baroreceptors which are largely expressed in the aortic arch. Also, increased load of the left heart may affect intracardiac ganglia. Repair of the CoA, especially following surgical intervention, may cause alteration in ANS function because of damage of nerves in the aortic arch. In term neonates with a simple coarctation, depressed baroreflex sensitivity, reduced HRV and increased blood pressure variability is found before intervention compared to healthy controls [77]. This cohort was studied again 5 years later after coactectomy and by that time, autonomic function seemed to have normalized [78]. One of the major clinical symptoms of these patients, even after successful repair of the coarctation is hypertension which is not fully understood. In patients after (successful) repair, hypertension continues to be a common complication with a median prevalence of 32.5% (range 25%–68%) [88]. Potential mechanisms explaining this postoperative hypertension include pathology of the vascular bed, local and systemic hemodynamic changes, shape of the aortic arch, impaired baroreceptor sensitivity and altered ANS activity. A potentially effective treatment being investigated for resistant hypertension is renal sympathetic denervation [89]. In a hypertensive group of six children who underwent repair of their coarctation at a later age (mean 9.9 years), diminished baroreceptor function was found 1–11 years after repair [80]. Kenny *et al.* [79] also found decreased BRS in hypertensive patients after repair compared to normotensive patients. There was no difference in BRS between normotensive patients and controls. Five years after surgery, BRS had normalized. However, the six patients included in this long term follow up were not hypertensive [78]. In a study comparing balloon angioplasty to surgical repair of the CoA, Choy *et al.* [81] found significant differences. Blood pressure in the group in which CoA was relieved by means of balloon angioplasty was lower compared to the surgical group. Plasma renin activity and catecholamines increased in the surgical group but not in the balloon angioplasty group. However, these neurohormonal changes were only measured before and shortly (1 and 2 days) after intervention. These findings suggest that ANS activity is already altered before intervention and seems to normalize in normotensive patients but not in hypertensive patients. Type of intervention may have an influence on ANS function late after repair.

### 5.5. Tetralogy of Fallot

Tetralogy of Fallot (TOF) is a congenital heart defect that encompasses stenosis of the pulmonary artery, ventricular septal defect, overriding aorta and right ventricular hypertrophy. Prevalence is about 4–5 in 10,000. In a group of postoperative TOF patients, Butera *et al.* [83] found a significant reduction in parasympathetic control measured by HRV from 24 hour Holter recording, particularly in patients with ventricular arrhythmia. Wyller *et al.* [84] found no significant differences in resting autonomic regulation measured by HRV between postoperative TOF patients and controls. However they did find a difference between controls and patients in response to the lower body negative pressure test; patients showed less HR increase and HRV increased whereas in controls lower body negative pressure led to a decrease in HRV. Silvilairat *et al*. [82] found a significant positive correlation between HF and exercise capacity (measured by maximal oxygen uptake) and between LF and exercise capacity. As exercise capacity is a predictor of mortality in this patient group, HRV can potentially be used as a proxy. In conclusion, results on HRV are inconclusive. One group does find aberrant ANS function and another does not while age does not differ much between study groups.

Massin *et al.* [59] described the biggest cohort and retrospectively studied a group of 258 children (56 of 258 children had been operated at least one year before) with various congenital heart defects. They grouped the cohort based on the New York Heart Association (NYHA) functional classes I–IV. Twenty four-hour holter analysis showed that all measures of both time and frequency domain HRV were decreased compared to controls in all groups except for NYHA class I. Interestingly, within the same NYHA class they found no differences between patients with different mechanisms causing their heart failure (e.g., volume overload, pressure overload, myocardial damage). There was no correlation between HRV and hemodynamics in this cohort. HRV is found to have a negative relationship with hospital stay duration [34,66] and reduced HRV can be restored with propranolol [63].

## 6. Conclusions

There is a dearth of research on pediatric cardiac autonomic nervous system function in congenital heart defects, especially before intervention and in relation to clinical outcome. Therefore, it is still uncertain whether altered cardiac autonomic nervous system control in these patients is part of the pathophysiology, a compensation mechanism, result of surgical procedures or a combination of these. The biggest cohort described concerning ANS function in pediatric congenital heart disease included 258 patients [59]. Unfortunately, this study was one of nine that did not segregate groups of different types of CHD or operated *versus* non operated patients in their analysis. Both are expected to be of influence on the function of the ANS. Overall, ANS function seems to be altered both before and after operation in children with a CHD. ANS control after catheter interventions may be more favourable compared to surgical intervention. However, large scale studies that measure both SNS and PNS for prolonged periods of time (e.g., 24 h) in well-defined patient cohorts in various phases of childhood and adolescence are currently lacking. Pending such studies there is not yet a good grasp on the extent and direction of PNS and SNS dysfunction in CHD.

ANS dysfunction might well play a clinically meaningful role in long term sequelae of patients with congenital heart disease. As in progressive left sided heart failure, compensatory changes in cardiac autonomic control may in time cause deterioration of heart function in these patients. Longitudinal studies linking cardiac autonomic control and clinical outcomes are warranted in order to gain more insight into the potentially causative role of the ANS in the etiology of CHD and the potential benefits of intervention targeting the ANS.

## Figures and Tables

**Table 1:** Heart rate variability (HRV) based measures. Ms; milliseconds.

HRV Variable (*Measure*)	Principle
Time domain measures	
*Mean RR*	Average of all NN intervals
*SDNN (ms)*	Standard deviation of all NN intervals (inter beat interval of two successive sinus beats)
*SDANN (ms)*	Standard deviation of the average NN intervals over a short period (typically 5 min) over the entire recording
*RMSSD (ms)*	Root mean square of successive differences between adjacent NN intervals
*Coefficient of variation*	Ratio of standard deviation of NN intervals and the mean of RR intervals
*NN50*	Number of pairs of successive NN intervals that differ >50 ms
*pNN50*	Proportion of NN50/total number of measured NN intervals
*pvRSA (ms)*	Respiratory sinus arrhythmia determined by peak-valley method. The shortest NN interval during inspiration is subtracted from the longest NN interval during exhalation (see Figure 2)
Frequency domain measures	
*HF (ms^2^)*	High frequency power. Power in the respiratory frequency range, typically from 0.15–0.4 Hz
*HFnu*	HF/(LF + HF)
*LF (ms^2^)*	Low frequency power. Power in the low frequency range, typically from 0.04–0.15 Hz
*LFnu*	LF/(LF + HF)
*LF/HF*	Ratio of low frequency power to high frequency power
*VLF (ms^2^)*	Very low frequency power. Power in the very low frequency range, typically from 0.003–0.04 Hz
*ULF (ms^2^)*	Ultra-low frequency power. Power in the ultra-low frequency range, typically <0.003 HZ
*TP (ms^2^)*	ULF + VLF + LF + HF

**Table 2:** Summary of preoperative, immediate postoperative and long term postoperative findings per congenital heart disease (CHD).

	Preop	Immediate Postop		Remote Postop	
ASD	HRV ↓ ^#,^*^,1^	Surgery	HRV ↓ (*vs.* preop)	Surgery	HRV ↑ (*vs.* preop)
		Cath	HRV ↑ (*vs.* preop)	Cath	HRV ↑ (*vs.* preop)
VSD	RSA ↑ (*vs.* ASD)	Cath	HRV ↓ in PH *vs.* NPP		No studies available
TGA	SNS innervation intact	Surgery	SNS denervation	Surgery	Reïnnervation SNS in 32% *^,2^
	HRV ↓ ^#^		No difference or lower HRV ^#,^*^,3^		PNS normalized; SNS ↓ ^#^
Uni ventricular heart	No studies available	Surgery	No studies available	Surgery	PNS ↓; sympathetic denervation; SNS ↑ ^#^
CoA	BRS & HRV ↓ ^#^	Surgery	SNS ↑ (*vs.* cath)	Surgery	HRV and BRS normalized
		Cath	No studies available	Cath	No studies available
TOF	No studies available	Surgery	No studies available	Surgery	HRV ↓ ^#^

Immediate postop <1 year after intervention; PH = Pulmonary Hypertension; NPP = Normotensive Pulmonary Pressure; ^#^ Compared to healthy controls; *^,1^ Found in all studies measuring 24 h HRV but not in one study measuring 15 min HRV; *^,2^ In patients operated within the first 55 days of life, almost all showed positive MIBG uptake compared to only half of patients operated later in life; *^,3^ Patients did show a slower recovery of HRV after feeding.

**Table 3:** Overview of studies grouped by type of CHD. Preop; preoperatively.

Author	Patient Group (N, Type CHD)	Age Patients	Pre or Postop	Control Group (N, Age)	Measure	Outcome Preoperatively	Outcome Postoperatively
Heragu *et al.* [34]	*N* = 36. Type of CHD not specified.	2 w–15 y	Preop & ± 1 w postop	*N* = 45. 3 w–16 y	24 h HRV	P *vs.* C: no difference in mean RR, SDNN, SDNN/RR, SDANN, LF and LF/HF. TP and HF ↓	All measures except for LF/HF ↓ compared to preop. LF/HF ↑ postop.
Massin *et al.* [59]	*N* = 258. Various types CHD.	2 d–14 y	Both	*N* = 210. 3 d–14 y	24 h HRV	SDNN, SDNNi, SDANNi, rMSSD, pNN50, LF, HF, LF/HF ↓ in pts in NYHA class II–IV.	SDNN, SDNNi, SDANNi, rMSSD, pNN50, LF, HF, LF/HF ↓ in pts in NYHA class II–IV.
Ohuchi *et al.* [58]	*N* = 143. ASD, VSD, RVOTR.	Mean 14.6 y	Preop & >1 m postop	*N* = 47. 15.6 ± 4	5 min HRV. Plasma NE. BRS, scintigraphy, blockade study.	-	P vs C: BRS, Log HF & Log LF, H/M↓. Plasma NE and =.
McGlone *et al.* [60]	*N* = 20. Various CHD	0–16 y	Preop & 1 d postop	No	1 h HRV preop, 24 h HVR postop	-	sNN50, SDNN, SDANN, RMSSD, SDNNi ↓ after surgery.
Currie *et al.* [61]	*N* = 12. TOF, CoA.	4 ± 1 y	Postop	*N* = 12. 5 ± 1 y	5 min HRV	-	P *vs.* C: SDRR, RMSSD, pNN50, log LF, LF, log HF, HF, LF/HF, DFA =.
Aletti *et al.* [62]	*N* = 15. Various CHD.	Mean 24 m	Preop	*N* = 10. 15 ± 10 m	10 min HRV	P *vs.* C: TP and LF ↑. Mean RR, VLF, HF =.	-
Buchorn *et al.* [63]	*N* = 14. Various CHD.	2.6 ± 1.9 m	Preop	*N* = 70. 2.1 ± 2.7 m	24 h HRV. Plasma NE and E.	P (standard treatment) *vs.* C: SDNN, SDANN, rMSSD, VLF, HF, LF ↓. Mean RR, pNN50, LF/HF, TP =. P (propanolol) *vs*. C: Mean RR ↑, VLF ↓. All other =.	-
Dzimir *et al.* [64]	*N* = 112. TOF, VSD, PST, COA, CPL.	Median 36 m	During catherization	*N* = 14. median 48 m	Plasma E&NE. α- and β adrenoreceptor activity	PST *vs.* C: plasma E and NE ↑. All groups except CoA increased α-adrenoreceptor densities. LRS & PST vs C: Β-adrenoreceptors ↓.	-
Goudjil *et al.* [65]	*N* = 22. PDA.	28 w	Preop	*N* = 22. 28 w	4 min HRV	P vs C: TP, LF, HF nu, HF/LF, SNDD ↓. HF, mean RR, RMSSD, SDSD =.	-
Kaltman *et al.* [66]	*N* = 60. Various CHD.	4.9 ± 3.3 y	Preop & 0–6 m postop	No	24 h HRV	Pts with biventricular *vs.* univentricular heart: mean HR, LF, LF, LF/HF =.	Pts with biventr *vs.* univentr heart: Mean HR ↓ LF ↑ at discharge. 3–6 m postop: Mean HR, LF, LF, LF/HF =.
Bakari *et al.* [54]	*N* = 28. ASD.	6.6 ± 2.1 y	Preop	*N* = 32. 6.4 ± 2.2 y	24 h HRV	P vs C: SDNN, SDANN, rMSSD, SD, SDNN index, PNN50, mean RR, TP, HF, LF/HF ↓.	-
Massin *et al.* [55]	*N* = 20. ASD.	Range 3–14 y	Preop	*N* = 210. 3 d–14 y	24 h HRV	P *vs.* C: mean RR, SDNNi, SDANNi, pNN50, LF, HF ↓.	-
Finley *et al.* [53]	*N* = 10. ASD.	Range 4–16 y	Preop & ± 5 m postop	*N* = 10. mean 6.1 y	10–15 min HRV and respiration standing and supine	P *vs.* C: Supine: mean RR, LF, resp rate =, SDNN, HF ↓. LF/HF ↑. Upright: mean RR, LF, HF, LF/H, resp rate =. SDNN ↓	P preop *vs.* P postop: Supine: mean RR, LF, HF, HF/LF =. SDNN ↑. Upright: mean RR, SDNN, LF, HF, HF/LF =.
Bialkowski *et al.* [56]	*N* = 19. ASD	2.5–14 y	Preop & 1 & 3 m postop	From earlier non-english publication	24 h HRV	P *vs.* C: SDNN, SDANN, SDNN index, rMSSD, pNN50 ↓.	P (preop) *vs.* P (after transcutaneous intervention): SDNN and SDANN ↑ 1m after intervention. SDNN, SDANN, SDNN index, rMSSD, pNN50 ↑ 3m after intervention. P (preop) *vs.* P (after surgical repair): SDNN, SDANN, SDNN index, rMSSD, pNN50 ↓ 1m postop. SDNN, SDANN index ↑ 3m postop.
Hata *et al.* [52]	*N* = 43. ASD, VSD.	ASD 4.6 ± 3.6 y; VSD 4.1 ± 6.4 y	Preop	No	HRV and respiration (time of recording not specified).	P (ASD) *vs.* P (VSD): HF, RSA ↓. Respiratory rate, LF, TP, LF/HF = .	-
Kul Yum *et al.* [57]	*N* = 32. VSD.	<12 months	Preop	No	5 min HRV during catherization	P (hypertemsive) *vs.* P (nonhypertensive) HR, SDNN =. LF and HF ↓.	-
Kondo *et al.* [67]	*N* = 51. TGA.	Mean 4.8 y	Preop & 1 m–10 y postop	*N* = 51. 4.2–6 m	Scintigraphy	All pts and controls showed positive MIBG uptake.	<1 m after ASO, all pts showed negative MIBG uptake. 15/47 pts negative MIBG uptake late postop.
Harrison *et al.* [68]	*N* = 15. TGA.	0–8 w	Preop & 2–8 w postop	*N* = 16. Age-matched.	2–4 h HRV	P *vs*. C: HF, LF ↓.	P *vs*. C: HF, LF =. Pts showed delayed recovery of HF after feeding.
Harrison *et al.* [69]	*N* = 15. TGA.	0–3 y	2 w and 3 y postop	*N* = 12. Age matched	15 m HRV	-	P *vs.* C: HF =. HF reactivity ↓.
Harrison *et al.* [70]	*N* = 15. TGA.	2–8 w	2–8 w postop	*N* = 16. Age matched	15 m HRV	-	P *vs.* C 2w postop: baseline HF, recovery HF ↓. P vs C 8w postop; baseline HF, recovery HF =.
Doksoz *et al.* [71]	*N* = 22. TGA.	Mean 59.5 ± 38.7 m	Postop	*N* = 22. Age 65.1 ± 39.4 m	24 h HRV	-	P *vs.* C: mean HR, max HR, min HR = . SDANN, VLF ↑. When awake, SDNN, rMSSD, pNN50, TP, VLF ↑. When asleep SDNN, rMSSD, pNN50, TP, VLF =.
Falkenberg *et al.* [72]	*N* = 8. TGA.	15.8 ± 1.5 y	15 y postop	*N* = 15. Age 19.7 ± 1 y	BRS and NE whole body and cardiac spillover	-	P *vs.* C: mean HR, BRS =. Total body NE spillover, regional spillover ↓.
Madan *et al.* [73]	*N* = 46. Univentricular heart.	BDG 5.2 ± 5.3 y Fontan 9.3 ± 5.9 y	Preop & 2 and 9 m postop	No	900 s HRV	P (BDG) *vs*. P (Fontan): coefficient of variance, SDNN, RNSSD, NN50, pNN50, VLF, HF, TP, LF/HF =.	P (BDG) *vs.* P (Fontan) 2 m postop: RMSSD ↑. P (BDG) 9m postop: coefficient of variance, TP, LF, VLF ↑. P (BDG) 9m postop: RMSSD, HF, LF, VLF ↑. P (BDG) *vs.* P (Fontan) 9 m postop: Coefficient of variation ↑.
Rydberg *et al.* [74]	*N* = 15. Univentricular heart.	Age not specified	Mean 7.2 y postop	No	24 h HRV. Poincaré plots.	-	P (with VA) *vs.* P (without VA): TP, VLF, LF, HF, HF/LF =. SD poincaré plots ↑.
Ohuchi *et al.* [75]	*N* = 63. Univentricular heart.	TCPC 12.8 ± 5.0 y, APC 14.2 ± 3.9 y	Postop	*N* = 44. 14.7 ± 3.9 y	Blockade study, scintigraphy, plasma NE	-	P *vs*. C: Log HF, log LF, BRS, H/M ↓. NE ↑.
Butera *et al.* [76]	*N* = 39. Univentricular heart.	Mean 12.2 ± 4.1 y	Mean 5.8 y postop	*N* = 18. 11.1 ± 2.5 y	24 h HRV	-	P *vs.* C: SDNN, RMSSD, pNN50, TP, LF, HF ↓.
Polson *et al.* [77]	*N* = 8. CoA.	Term neonates	Preop	*N* = 13. term neonates.	Spontaneous BRS, 15 min HRV, BPV	P *vs*. C: HR =. BRS ↓. SDNN, RMSSD, TP, LF, HF ↓. BPV HF ↑.	-
Kenny *et al.* [78]	*N* = 6. CoA.	5 y	Postop	*N* = 7. Age & sex matched	Spontaneous BRS, 15 min HRV, BPV		P *vs.* C: BRS, SDNN, TP, LF, HF, BPV =.
Kenny *et al.* [79]	*N* = 29. CoA.	14-18 y	<1 y posop	*N* = 20. 15.7 ± 0.3 y	BRS, HRV, BPV		P *vs.* C: HR, HF, LF, BPV =.
Beekman *et al.* [80]	*N* = 6. CoA.	Mean 16.8 ± 2.0 y	1–11 y postop	*N* = 7. 15.4 ± 4 y	BP, HR response to exercise, BRS	-	P *vs*. C: BRS ↓. BP response to exercise ↑. Resting HR, max HR =.
Choy *et al.* [81]	*N* = 15. CoA.	Mean 8.5 y	Preop & <1 y postop	No	BP, Plasma renin and catecholamine		P (surgical intervention) *vs.* P (cathetherization): plasma renin, plasma catecholamines ↑.
Silvilairat *et al.* [82]	*N* = 30. TOF.	Median 14 y	2–16 y postop	No	24 h HRV	-	Positive correlation between LF and VO2peak and HF and VO2peak.
Butera *et al.* [83]	*N* = 23. TOF.	14 ± 6.6 y	Postop	*N* = 18. 11.2 ± 4.9 y	24 h HRV	-	P *vs.* C: SDNN, RMSSD, pNN50, TP, LF, HF, LF/HF ↓.
Wyller *et al.* [84]	*N* = 17. TOF.	Median 16 y	10–16 y postop	*N* = 56. Age range 13–18 y	24 h HRV, LBNP	-	P *vs.* C during rest: LF, HF, LF/HF, SDNN, pNN50, RMSSD =. P *vs.* C during LBNP: HR response ↓. TP, HF, SDNN, pNN50, RMSSD ↓ in controls and ↑ in pts.

Postop; postoperatively. w; weeks. m; months. Y; years; pts; patients. P *vs.* C; patient *versus* control.

## Abbreviations

The following abbreviations are used in this manuscript:

Ach	acetylcholine
ANS	autonomic nervous system
ASD	atrial septal defect
ASO	arterial switch operation
BDG	bidirectional Glenn shunt
BP	blood pressure
BPV	blood pressure variability
BRS	baroreceptor sensitivity
CHD	congenital heart disease
CoA	coarctation of the aorta
CPL	complex cyanotic heart disease
DFA	detrended fluctuation analysis
E	epinephrine HRV—heart rate variability
LBNT	low body negative pressure test
LRS	left to right shunt
MIGB	metaiodobenzylguanidine
NE	norepinephrine
NN interval	normal-to-normal interbeat interval
NYHA	New York Heart Assosiation
PEP	pre ejection period
PNS	parasympathetic nervous system
PST	pulmonary stenosis
Qp/Qs	pulmonary to systemic flow ratio
SD	standard deviation
SNS	sympathetic nervous system
TGA	transposition of the great arteries
TOF	tetralogy of Fallot
VA	ventricular arrhythmia
VSD	ventricle septal defect
